# Supplementary material for: Values and Risk Perception Shape Canadian Dairy Farmers’ Attitudes toward Prudent Use of Antimicrobials
Source: Antibiotics (Basel). 2022 Apr 20;11(5):550. doi: 10.3390/antibiotics11050550 (PMC9137716; doi:10.3390/antibiotics11050550)
Supplement: Supplementary file 1 [file antibiotics-11-00550-s001.zip › antibiotics-1690931-supplementary.pdf]

Values and risk perception shape Canadian dairy farmers' attitudes toward prudent use of antimicrobials

Supplementary file 1. Questions included in the analysis

Individual Demographics

1. Are you currently dairy farming in Ontario?
   - Yes
   - No

2. Please select the option that best describes your role on the farm:
   - Farm Owner
   - Manager
   - Family member of owner
   - Employee (other than manager)- Non-family of owners
   - Other (specify)

3. Please indicate the age category you belong to:
   - < 20 years
   - 20 to 29 years
   - 30 to 39 years
   - 40 to 49 years
   - 50 to 59 years
   - 60 to 69 years
   - > 70 years

4. Which of the following best describes your HIGHEST level of education?
   - Some public school
   - Completed public school
   - Some high school
   - Completed high school
   - Apprenticeship training and trades
   - Completed College
   - Completed University
   - Completed graduate education
   - Professional degree

HOW MUCH LIKE YOU IS THIS PERSON?

5. Please indicate the gender pronoun with which you identify (this information is used to tailor the questions in the following section).
   - He/him
   - She/her
   - They/them
   - I prefer not to say

Here we briefly describe some people. Please read each description and think about how much each person is or is not like you. Using a 6-point scale from “not like me at all” to “very much like me,” choose how similar the person is to you.

6	5	4	3	2	1
Very much like me	Like me	Somewhat like me	A little like me	Not like me	Not like me at all

_____ 1. S/he believes s/he should always show respect to his/her parents and to older people. It is important to him/her to be obedient
_____ 2. Religious belief is important to him/her. S/he tries hard to do what his religion requires.
_____ 3. It's very important to him/her to help the people around him/her. S/he wants to care for their well-being.
_____ 4. S/he thinks it is important that every person in the world be treated equally. S/he believes everyone should have equal opportunities in life.
_____ 5. S/he thinks it's important to be interested in things. S/he likes to be curious and to try to understand all sorts of things.
_____ 6. S/he likes to take risks. S/he is always looking for adventures.
_____ 7. S/he seeks every chance he can to have fun. It is important to him/her to do things that give him/her pleasure.
_____ 8. Getting ahead in life is important to him/her. S/he strives to do better than others.
_____ 9. S/he always wants to be the one who makes the decisions. S/he likes to be the leader.
_____ 10. It is important to him/her that things be organized and clean. S/he really does not like things to be a mess.
_____ 11. It is important to him/her to always behave properly. S/he wants to avoid doing anything people would say is wrong.
_____ 12. S/he thinks it is best to do things in traditional ways. It is important to him/her to keep up the customs s/he has learned.
_____ 13. It is important to him/her to respond to the needs of others. S/he tries to support those s/he knows.
_____ 14. S/he believes all the worlds' people should live in harmony. Promoting peace among all groups in the world is important to him/her.
_____ 15. Thinking up new ideas and being creative is important to him/her. S/he likes to do things in his/her own original way.
_____ 16. S/he thinks it is important to do lots of different things in life. S/he always looks for new things to try.
_____ 17. S/he really wants to enjoy life. Having a good time is very important to him/her.
_____ 18. Being very successful is important to him/her. S/he likes to impress other people.
_____ 19. It is important to him/her to be in charge and tell others what to do. S/he wants people to do what s/he says.
_____ 20. Having a stable government is important to him/her. S/he is concerned that the social order be protected.

General Farm Demographics

6. How many cows are you MILKING in your herd today?

7. What percentage of the dairy herd are the following breeds?

**Percentage of herd**	**Breed**
	Holstein
	Jersey
	Other (specify)

8. During the 12-month period between January 1, 2019 and December 31, 2019, what was the average milk production in kg per cow (305 days)?

9. Please choose the type of barn in which you house most (>50%) of your MILKING cows
   - o Free stall barn
   - o Tie stall barn
   - o Bedded pack
   - o Other (specify)

10. What best describes the system you use to milk the majority (>50%) of your herd?
    - o Pipe-line
    - o Parlour
    - o Robotic milking system
    - o Other (Specify)

11. Is your farm certified as an organic dairy farm?
    - o Yes
    - o No

12. Indicate the frequency of regularly scheduled visits with the herd veterinarian (e.g. Herd Health)?
    - o Weekly
    - o Every 2 weeks
    - o Every 3 weeks
    - o Monthly
    - o Every 2 months
    - o Less frequent than every 2 months
    - o The farm does not have regularly scheduled visits with a veterinarian

<u>Please answer the following questions as they pertain to antimicrobial stewardship</u>

13. Are you involved with the antimicrobial treatment of animals on the farm? [D]
    - o Yes – answer the questions below
    - o No – go to the final section of the survey

We are interested in how you use antibiotics in your dairy animals. Antibiotics (also called antimicrobials) are medicines used to treat infections and diseases caused by bacteria. These include drugs like penicillin, tetracycline, or ceftiofur. They are typically given by injection or as tubes in the udder.

14. Do you have an SOP for antibiotic drug use for common diseases on your farm?
    - Yes
    - No

15. Thinking of the last 10 times you treated an animal with antibiotics on your farm, for how many did you follow the written SOP?
    - None
    - 1 to 3
    - 4 to 6
    - 7 to 9
    - All 10

16. Once you have made the decision to treat with an antibiotic, what are the most important reasons for you to select a particular antibiotic product? Select the 5 most important to you and rank them in order from the most important to less important.
    - My own experience with similar cases
    - Previous recommendations from my veterinarian
    - Consultation with my veterinarian about the specific case
    - Other producers' advice
    - My farm's written protocol for the disease
    - Price of the product
    - The length of the withholding period
    - Whether I am under or over quota
    - Ease of administration
    - Number of doses required
    - Having an on-label indication for the condition I am treating
    - Potential development of antibiotic resistance
    - Other (specify)

17. What proportion of all the antibiotic treatments on your farm are recorded (either in your computer or in a logbook)?
    - <50%
    - 50 to 75%
    - 75 to 98%
    - 98-100%

18. If you do not record some antibiotic treatments, please rank the reasons in order from the most common to the least common reason for you not to record antibiotic treatments.
    - I only record treatments that have a milk withdrawal
    - I only record treatments in lactating cows
    - I forget to write things down because I'm busy
    - It's inconvenient to record treatments
    - I choose not to record treatments

19. How often do you contact your veterinarian before using antibiotics in your farm?
    - Never
    - Sometimes
    - Often

- Always

20. Which of the following, if any, prevents you from reaching out to your veterinarian more regularly?
    - Finance
    - Comfort level
    - Relationship
    - Time
    - I feel the veterinarian will disagree with me

21. Which factors are most important to you when deciding whether to use an antibiotic treatment in <u>lactating cows</u>? (Rank your top three in order from the most important to less important)
    - Cow body temperature
    - Whether the cow appears dull or active
    - Response to non-antibiotic therapy given first (e.g. calcium, fluids or anti-inflammatory medication)
    - Time since the sign or symptom started
    - Age or lactation number of the cow
    - Days in milk of the cow
    - Whether I am over or under quota
    - Whether there are quota incentive days that I could fill
    - Previous antibiotic treatments in the same cow this lactation
    - Other (specify)

22. What are the most common reasons for which you treat with antibiotics in <u>lactating cows</u>? (Rank your top three in order from the most important to less important)
    - Pneumonia
    - Lameness
    - Diarrhea
    - Mastitis (excluding dry cow therapy)
    - Fever (not as part of a disease listed here)
    - Post-surgical and wound management
    - Uterine infections (metritis or endometritis)
    - Other (specify)

23. Which injectable antibiotics or products do you use most frequently in lactating cows? (Rank your top three in order based on most frequently used in lactating cows in the last 12 months)
    - Penicillin (Depocillin, ProPen)
    - TMS (Borgal, Trivetrin, Timadox)
    - Ceftiofur (Excenel, Excede, Ceftiocyl)
    - Tetracycline (Liquamycin, OxyVet)
    - Other (give drug or product name)

24. What are the most common reasons you usually treat with antibiotics in <u>pre-weaned calves</u>? (Rank your top three in order from the most important to less important over the last 12 months)
    - Pneumonia
    - Diarrhea (scours)
    - Navel (umbilical) infections
    - Arthritis
    - Other (specify)

25. Which antibiotics or product do you use most frequently in calves under 3 months old? (Rank top three in order of most frequently used to least frequently used in your calves over the last 12 months)
    - Florfenicol (Nuflor, Resflor)
    - Mactolide drugs (Draxxin, Zactran, Zuprevo)
    - Tilmicosin (Micotil)
    - Ceftiofur (Excenel, Excede, Ceftiocycl)
    - Penicillin (Depocillink ProPen)
    - Tetracycline (Liquamycin, OxyVet)
    - Fluorquinolones (Baytril, A180)
    - TMS (Borgal, Trivetrin, Timadox)
    - Other (give drug or product name)

26. If you see a calf that is standing and has scours (diarrhea) do you normally administer antibiotic treatment right away to all such cases?
    - Yes
    - No (skip next question)

27. Which factors do you consider before administering antibiotic treatment to your calves with scours? (Select all that apply)
    - Appearance of the manure (how watery)
    - Attitude of the calf (how dull or inactive it looks)
    - Level of dehydration (sunken eyes)
    - Fever
    - How well the calf is eating
    - Other (specify)

28. If you see a calf with respiratory symptoms (coughing, difficulty breathing) do you normally administer antibiotic treatment right away to all such cases?
    - Yes
    - No (skip next question)

29. Which factors do you consider before administering antibiotic treatment to your calves with respiratory disease? (Select all that apply)
    - Presence of nasal or eye discharge
    - Frequency of coughing
    - Elevated breathing or respiratory rate
    - Attitude of the calf (how dull or inactive it looks)
    - Fever
    - How well the calf is eating
    - Other (specify)

30. In general, what are the most important factors you consider when selecting a particular antibiotic product for calves? Rank your top three in order of importance
    - My own experience with similar cases
    - Previous recommendations from my veterinarian
    - Consultation with my veterinarian about the specific case
    - Other producer’s advice
    - My farm’s written protocol for the disease
    - Price of the antibiotic product
    - The length of the drug withdrawal time

- Ease of administration
- Number of doses required
- Having an on-label indication for the condition I am treating
- Potential development of antibiotic resistance
- Other (specify)

For the following questions, antibiotic resistance means that bacteria become resistant to the medications used to treat disease, so that the antibiotic medication does not work as well or does not work at all.

31. Please indicate your level of agreement with the following statements.

	Strongly disagree	Disagree	Neither agree nor disagree	Agree	Strongly agree
There is overuse of antibiotics in dairy production	☐	☐	☐	☐	☐
I could explain what antibiotic resistance is to my neighbor	☐	☐	☐	☐	☐
Antibiotic resistant infections are NOT an important problem in dairy cattle	☐	☐	☐	☐	☐
We should reduce the use of antibiotics in dairy production	☐	☐	☐	☐	☐
Milk production will be reduced if antibiotic use is decreased	☐	☐	☐	☐	☐
Animal welfare would be worse if antibiotic use is decreased	☐	☐	☐	☐	☐
The use of antibiotics on my farm could cause antibiotic resistance on my farm	☐	☐	☐	☐	☐
The use antibiotics on my farm could cause antibiotic resistance on other farms	☐	☐	☐	☐	☐
The use of antibiotics on my farm could cause antibiotic resistance in humans	☐	☐	☐	☐	☐
If I knew which antibiotics are most important for human medicine, I would avoid using them in my cattle	☐	☐	☐	☐	☐
When I treat an animal, I think about the risk of antibiotic resistance in cattle	☐	☐	☐	☐	☐

When I treat an animal, I think about the risk of antibiotic resistance in humans	☐	☐	☐	☐	☐
My veterinarian encourages me to use antibiotics	☐	☐	☐	☐	☐
My veterinarian does not know how I use antibiotics in my animals	☐	☐	☐	☐	☐
I have had a discussion with my veterinarian about when and how to use antibiotics for treatments when I do not call the vet	☐	☐	☐	☐	☐
I follow written protocols or the product label exactly	☐	☐	☐	☐	☐
Newer antibiotics are more effective than older ones	☐	☐	☐	☐	☐
Antibiotics with no milk withholding time are less likely to cause antibiotic resistance than those that require a withdrawal period	☐	☐	☐	☐	☐
Antibiotic use in humans (prescribed by doctors) is the main cause of antibiotic resistance in humans	☐	☐	☐	☐	☐
Antibiotic resistant infections in people are an important problem	☐	☐	☐	☐	☐
When I, as a patient, use antibiotics, I follow the prescription exactly	☐	☐	☐	☐	☐
I am satisfied with my veterinarian					

32. Should there be more initiatives to promote responsible use of antibiotics in the dairy industry?

   - Yes
   - No (skip next question)

33. If yes, select the one statement that best fits your opinion.
   - Put more regulations in place to change or reduce use of antibiotics
   - Provide more education or promotion to change or reduce use
   - Measure use and provide benchmarking to compare use among dairy farms
   - Provide incentives to change or reduce use

34. In your view, who should take the lead on promoting responsible use of antibiotics in the dairy industry?
   - International organizations
   - My national government
   - My provincial government
   - Dairy Farmers of Ontario
   - Milk processors
   - Retailers or restaurants
   - Pharmaceutical or animal health companies
   - Veterinarians
   - Individual farms

- No one – no changes are needed

35. In your opinion, what group of animals it would be easiest to reduce antibiotic use without causing harm? Rank in order form easiest to most difficult
    - Pre-weaned calves
    - Weaned calves and pre-breeding heifers
    - Breeding age and pregnant heifers
    - Lactating cows
    - Dry cows
